# Achieving COVID‐19 herd immunity in Bangladesh

**DOI:** 10.1002/puh2.97

**Published:** 2023-06-06

**Authors:** Rabeya Mashferat Mary, Falguni Alam, Adriana Viola Miranda, Don Eliseo Lucero‐Prisno, Md. Mofijul Islam Bulbul

**Affiliations:** ^1^ Shaheed Monsur Ali Medical College & Hospital Dhaka Bangladesh; ^2^ Department of Public Health University of Chester Chester UK; ^3^ International Centre for Diarrhoeal Disease Research (icddr,b) Dhaka Bangladesh; ^4^ Department of Public Health North South University Dhaka Bangladesh; ^5^ Global Health Focus Asia Bandung Indonesia; ^6^ Department of Global Health and Development London School of Hygiene and Tropical Medicine London UK; ^7^ Faculty of Management and Development Studies University of the Philippines Open University Los Baños Laguna Philippines; ^8^ Faculty of Public Health Mahidol University Bangkok Thailand; ^9^ Program Manager NNS Ministry of Health and Family Welfare Dhaka Bangladesh

**Keywords:** Bangladesh, COVID‐19, displaced population, herd immunity, pandemic, refugees, Rohingya, vaccination

## Abstract

Achieving herd immunity against COVID‐19 through vaccination is a global target, including in lower resource settings. Despite the challenges and limitations, Bangladesh has achieved its target of vaccinating 70% of its population, with good vaccine coverage among refugees, remote population, and women. This can be attributed to the evidence‐informed adaptability and collaborative approaches of the program. Yet some challenges remain, including dependence on donors and the need to ramp up vaccination among the underserved communities. This article discusses the factors contributing to the achievements of the COVID‐19 vaccination program of Bangladesh and the remaining challenges that should be addressed by the government to ensure the sustainability of the program and inform future vaccination efforts.

## BACKGROUND

The COVID‐19 began spreading in December 2019 resulting in the COVID‐19 pandemic. In Bangladesh, the first COVID‐19 case was identified on 8 March 2020. As of 31 January 2023, 2037,478 confirmed cases and 29,441 deaths have been reported [1]. Bangladesh faces numerous issues in effectively controlling the COVID‐19 pandemic due to various factors—population living in densely packed areas, widespread misinformation, and the ongoing humanitarian crisis with the influx of Forcibly Displaced Myanmar Nationals (FDMN or Rohingya refugees) [2]. As of December 2022, 952,309 refugees live in camps across the country, of which most live in Cox's Bazar, the largest refugee camp globally [3].

Bangladesh follows the global WHO vaccination target of vaccinating at least 70% of its population to achieve herd immunity. In the beginning of June 2021, less than 4% of the total population received both vaccine doses. A year and a half later (31 January 2023), the two‐dose vaccination rate has risen to 78.86%, reaching the intended target despite challenges as a low‐ and middle‐income country. All eight divisions within Bangladesh have already achieved the targeted 70% vaccination coverage. Booster doses have been given to approximately 49.14% of the population [4]. The country's vaccination program has major achievements in terms of its vaccination coverage—the male and female vaccination rates are almost comparable, and almost all FDMN have been vaccinated [4, 5]. Nonetheless, several challenges remain. This commentary aims to understand the efforts of Bangladesh to achieve herd immunity through an equitable vaccination program and identify the remaining challenges that may hinder these efforts.

## COVID‐19 VACCINATION INITIATIVES

Initially, the government intended to vaccinate approximately 138.2 million people (80% of the population). The target was later lowered to 70%. The government started the COVID‐19 vaccination program on 27 January 2021, targeting only healthcare providers, and opened a national vaccination campaign on 7 February of the same year [1]. The vaccination is provided free of charge.

Seven vaccines are being used in the country: AstraZeneca is the most used vaccine, followed by Pfizer, Sinopharm, Moderna, Sinovac, Janssen (Johnson & Johnson), and Pfizer‐PF (Comirnaty), respectively [1]. Data from September 2022 reported that Bangladesh has secured vaccine supply for 269.11% of its population (approximately 450 million doses), of which 368.5 million doses have been delivered [6]. The government received various funding and international loan packages to purchase and procure vaccines, expand storage facilities, and distribute and deploy the vaccines [2, 6]. The COVAX facility has sent over 190 million doses, equivalent to 62% doses received by the country. Overall, the facility pledged to send Bangladesh vaccine doses amounting to approximately 160% of the population. The sources of these COVAX vaccines include donations from many donor countries [6]. To register, vaccinate, and monitor the vaccination efforts, a mandatory app‐based registration (the Surokkha app) system was developed [1].

From the start of the vaccination campaign, the government has promoted awareness through print and electronic media, the national COVID helpline, and by the community and religious leaders and healthcare providers. Volunteers from NGOs are doing home visits to debunk myths, help people find vaccination facilities, and assist in the vaccine registration process [7].

In the beginning of the vaccination program, FDMN were not included. This situation changed in early February 2021, when a revised version of the National Deployment and Vaccination Plan (NDVP) that included the FDMN population was published by the government. This inclusion has been the target of the Health Sector in Cox's Bazar, comprising international NGOs and UN bodies and led by the WHO [8]. The WHO played a crucial role in assisting the government in developing the NDVP [8, 9] and in upscaling vaccine supplies by advocating for vaccine donations to the COVAX facility [10].

## LESSONS LEARNED

The Bangladesh vaccination program strives to vaccinate a big percentage of the eligible population to achieve herd immunity. This is supported by initial microplanning, periodic assessments, evidence‐informed program improvements, and multi‐sectoral collaboration.

The continuous collaboration between the government and the civil society has allowed the program to reach the most remote areas. For instance, in June 2021, the government asked CARE, an NGO, to focus on Khulna, which is the second least vaccinated district in Bangladesh. The request included reaching one of the hardest to reach remote coastal areas. The NGO helped identify and address the main vaccination barriers, such as difficulties in registering online. Due to the success in Khulna, the government waived online registration for its mobile vaccination campaign [7].

Vaccination programs also have a focused target on refugees. As per the Bangladesh NDVP with the support of UN bodies and other humanitarian partners, a total of 56 health facilities have been working to vaccinate FDMN [9]. As of 4 December 2022, almost all FDMN have been fully vaccinated. At Cox's Bazar, 90% of the FDMN aged ≥12 years have been vaccinated, with 86% having received booster doses. Due to this vaccination effort, the trend of COVID‐19 cases among FDMN is decreasing, similar to the decreasing trend experienced by the Bangladeshi community [5]. The culturally sensitive awareness programs conducted in the refugee camp have also helped reduce vaccine hesitancy among the FDMN [10].

The real‐world inclusion of FDMN in the national vaccination program is not commonly seen in other countries. According to the IOM, although 30 out of 40 Asia‐Pacific countries included refugees in their national vaccination plan, in practice, vaccine accessibility is only experienced by refugees in 25 countries. Seven main barriers were identified globally: policy, financial barriers, technical barriers, informational barriers (including language, cultural, and trust barriers), lack of vaccine availability, logistical barriers, and discrimination [11]. In Bangladesh, these barriers were addressed due to the close coordination among NGOs and UN bodies. Vaccine hesitancy is being addressed by the collaboration of local community health workers and UN bodies, which visit camp houses weekly to educate FDMN about COVID‐19 and encourage them to be vaccinated [9].

In the beginning of the vaccination program, gender disparity was evident, with only 38% of the vaccine recipients being women [12]. It is also reflected in a 2020 survey that reported 60.5% of Bangladeshi women were not confident about their access to COVID‐19 vaccine, compared to 55.4% of men [13]. Now, the ratio of male to female vaccination rate is 1:1. This can be attributed to the free vaccination program and women‐specific awareness programs. Vaccine registrations were expanded beyond digital applications, considering that many women do not have access to mobile phones. Instead, door‐to‐door awareness programs were conducted [12]. From Bangladesh's experience, it is clear that the inclusion of marginalized communities, cross‐sectoral collaboration, including with international organizations, and the utilization of an appropriate mix of digitalized and conventional, culturally sensitive efforts are crucial to ensure widespread COVID‐19 vaccination.

## REMAINING CHALLENGES

Several challenges to the vaccination efforts in Bangladesh remain. These include the limited vaccination coverage of the indigenous population, the elderly, and hijra. Dependence on donors also remains an issue. The indigenous population, comprising 1%–2% of the total population, mainly lives in the Chittagong Hill tracts, remains left behind in the vaccination program. Although the data on vaccine inequity among the indigenous population is scarce, some issues have been reported: from 100 elderly residing in Battiapara, a village of the indigenous Mro tribe, only three have been vaccinated. This largely stems from misinformation about vaccine safety and religion‐related vaccine hesitancy: their religion, Krama, prohibits all forms of vaccinations. COVID‐19‐related awareness campaigns have mostly been done in the Bangladeshi national language despite the huge potential targeted awareness programs; among the Garo community, these programs have increased vaccine uptake to nearly 50% [14].

The transgender population (called *hijra* in Bangladesh) also lags in the vaccination coverage. Although there are approximately 10,000 people registered as hijra in the government database, only 608 are reported to have received the second dose in the national vaccination dashboard. This is explained by the lack of access and discrimination faced by hijra during the COVID‐19 pandemic and/or unstandardized reporting (in the government dashboard, only the second dose coverage report includes hijra as its metrics) [1, 15]. Many hijras do not have any birth certificate or national identity card, hindering the vaccination registration process. Furthermore, in the Surokkha app, there is no separate category for hijra, causing hijras to feel left out in the vaccination program. Some hijras have also expressed their fear over being vaccinated, showing the need for awareness programs that specifically target this community [16].

The vaccination rate among the elderly is lower than other age groups. The most recent data available on elderly, published in early 2022, reported that 60% of those aged ≥60 years have yet to be vaccinated [17]. Citizens aged over 60 who are incapable of independent movement and with disability or bed‐bound by illness are unable to reach vaccination sites.

Bangladesh's vaccine supply comes from donors and international funders. This poses issues to sustainability, particularly considering that new COVID‐19 variants are continuously emerging, necessitating booster shots. Currently, an agreement of coproducing Sinopharm locally in Bangladesh is in process. In addition, a private company named Globe Biotech is developing a COVID‐19 vaccine named BANGAVAX. The Bangladesh Medical Research Council (BMRC) has granted ethical clearance conditionally for human trials for this vaccine [18]. The impact of this effort remains to be seen.

## CONCLUSION

The experience of the Bangladeshi vaccination program shows that low‐resource settings are capable of achieving a good vaccination coverage employing real‐world evidence and assistance from various sectors. It is imperative that international organizations should actively partner with other stakeholders, including NGOs and UN bodies, and forge international collaborations with other countries.

It is also imperative that countries should continuously improve their COVID‐19 vaccination programs. Bangladesh should step up its efforts to ensure that no one is left behind in their vaccination programs. Engaging the indigenous population, hijra community, and the elderly should remain a priority. To reach the indigenous populations, awareness programs should be conducted using local languages with culture‐sensitive approaches. Awareness programs should also be conducted to reduce discrimination among the hijra community. Standardized reporting of the hijra community's vaccination rate is important to properly identify and address specific barriers faced by the community. Door‐to‐door awareness programs that have been created for women can be extended to cover for these communities including the elderly. To reduce reliance on donors, the effort of producing vaccines should be supported so that the country can improve the sustainability of its COVID‐19 vaccination program for years to come.

## AUTHOR CONTRIBUTIONS

*Data curation; writing—original draft*: Rabeya Mashferat Mary, Falguni Alam. *Conceptualization; data curation; writing—review and editing*: Adriana Viola Miranda, Don Eliseo Lucero‐Prisno III. *Writing—review and editing*: Md. Mofijul Islam Bulbul.

## CONFLICT OF INTEREST STATEMENT

AVM and DELP are editorial board members of the journal. They were excluded and blinded from all stages of the peer review of this manuscript.
